# Assembly of the Transmembrane Domain of *E. coli* PhoQ Histidine Kinase: Implications for Signal Transduction from Molecular Simulations

**DOI:** 10.1371/journal.pcbi.1002878

**Published:** 2013-01-24

**Authors:** Thomas Lemmin, Cinque S. Soto, Graham Clinthorne, William F. DeGrado, Matteo Dal Peraro

**Affiliations:** 1 Laboratory for Biomolecular Modeling, Institute of Bioengineering, School of Life Sciences, Ecole Polytechnique Fédérale de Lausanne (EPFL), Lausanne, Switzerland; 2 Department of Biochemistry and Biophysics, University of Pennsylvania, School of Medicine, Philadelphia, Pennsylvania, United States of America; 3 Department of Pharmaceutical Chemistry, University of California – San Francisco, San Francisco, California, United States of America; UNC Charlotte, United States of America

## Abstract

The PhoQP two-component system is a signaling complex essential for bacterial virulence and cationic antimicrobial peptide resistance. PhoQ is the histidine kinase chemoreceptor of this tandem machine and assembles in a homodimer conformation spanning the bacterial inner membrane. Currently, a full understanding of the PhoQ signal transduction is hindered by the lack of a complete atomistic structure. In this study, an atomistic model of the key transmembrane (TM) domain is assembled by using molecular simulations, guided by experimental cross-linking data. The formation of a polar pocket involving Asn202 in the lumen of the tetrameric TM bundle is crucial for the assembly and solvation of the domain. Moreover, a concerted displacement of the TM helices at the periplasmic side is found to modulate a rotation at the cytoplasmic end, supporting the transduction of the chemical signal through a combination of scissoring and rotational movement of the TM helices.

## Author Summary

Two-component systems (TCSs) are signaling complexes essential for bacterial survival and virulence. PhoQ is the histidine kinase chemoreceptor of the PhoQ-PhoP tandem machine that detects the concentration of cationic species at the inner membrane of Gram-negative bacteria. A full understanding of the PhoQ signal transduction mechanism is currently hindered by the lack of a complete atomistic structure. Here, by using molecular simulations integrated with cross-linking disulfide scanning data, we present the first structural model of the transmembrane (TM) portion of PhoQ from *E. coli*. Its structural and dynamic features induce a concerted displacement of the TM helices at the periplasmic side, which modulates a rotation at the cytoplasmic end. This supports the idea that signal transduction is promoted through a combination of scissoring and rotational movements of the TM helices. This complex mechanism is the key to understanding how the chemical stimuli sensed by the periplasmic sensor domain trigger, via the relay of the HAMP domain, the histidine auto-phosphorylation and kinase/phosphatase activity at the cytoplasmic end.

## Introduction

Two-component systems (TCS) are protein signaling complexes present in most species of bacteria and are used to sense a wide range of environmental stimuli and couple them to adaptive responses [1]. The structure of a prototypical TCS consists of a membrane-spanning histidine kinase sensor, that senses the stimuli at the periplasmic region, and activates a cytoplasmic response regulator. The PhoQP TCS is reported to play a role in the defensive and virulence mechanism for certain Gram-negative bacteria [2], [3]. External stimuli, such as the presence of antimicrobial peptides at the periplasmic surface, lead to the auto-phosphorylation of the PhoQ histidine kinase core, and to the subsequent transfer of the phosphoryl group to the response regulator, which elicits the regulatory response (kinase activity). The phosphorylated PhoP promotes the transcription of genes which leads to the modification of the outer membrane. These modifications increase the synthesis of enzymes that deacylate, almitoylate, palmitoylate, hydroxylate, and attach aminoarabinose to lipid A, thus promoting bacterial resistance to antimicrobial peptides (AMP) and reducing the host recognition of lipid A [4]. Only a limited number of two-component systems are found in a few eukaryotes [5]. Thus, apart from the basic understanding of the fundamental signaling mechanism, PhoQP TCS is a promising target for the development of synthetic antimicrobial drugs.

**Figure 1:**
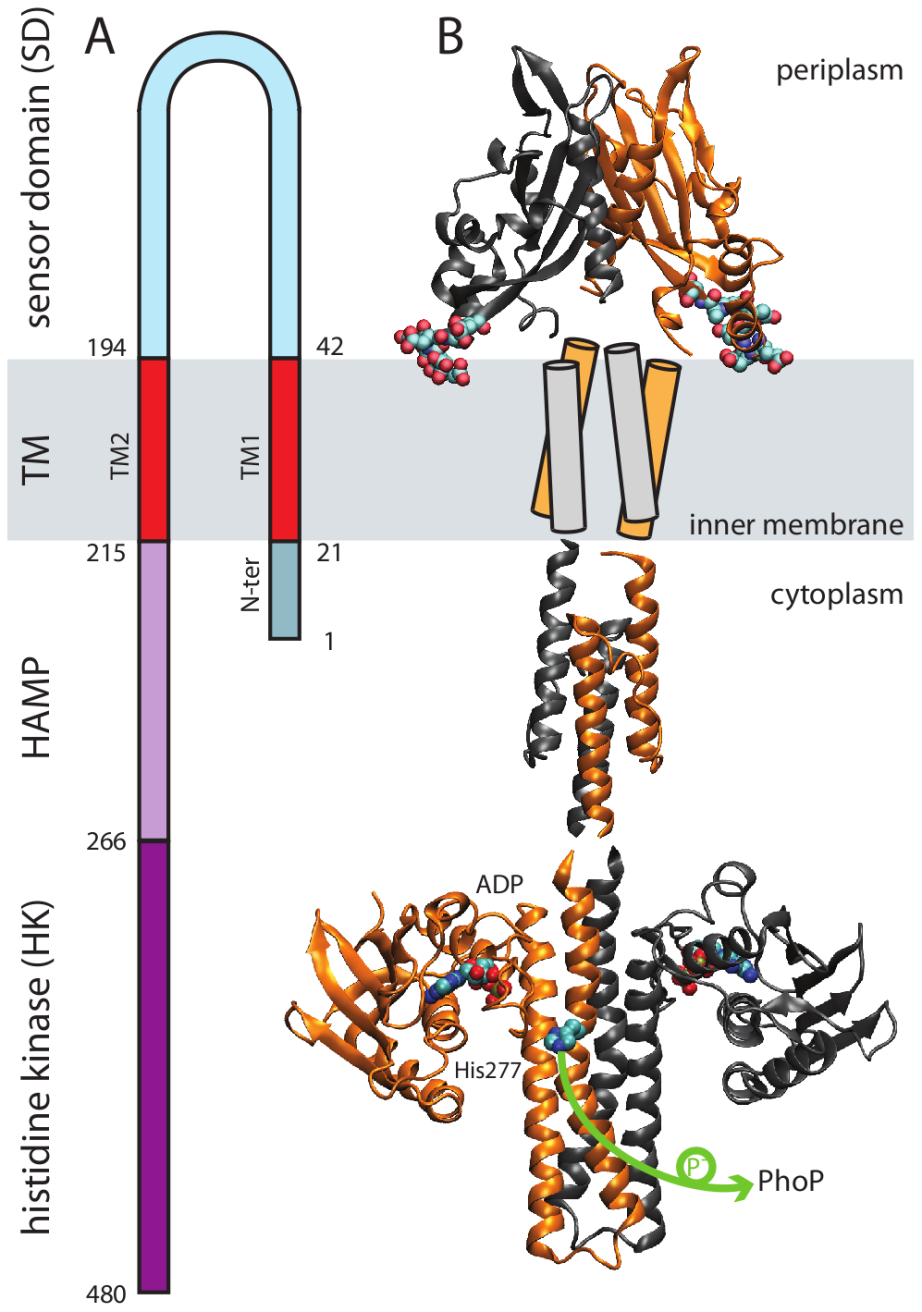
Schematic representation of the PhoQ histidine kinase in two-component systems. (A) The sequence topology of PhoQ is related to its (B) 3D homo-dimer schematic arrangement. The following available structures are used to model the global PhoQ structure: the sensor domain solved from *E. coli* (PDB code 3BQ8 [9]); the cytoplasmic HAMP NMR structure from *Archaeoglobus fulgidus* (PDB code 2L7H [30]), and the histidine kinase crystal structure from *Thermotoga maritima* (PDB code 2C2A [58]). The sensor domain harbors several acidic residues (148—EDDDDAE—154, shown in space-filled representation). They have been proposed to be involved in the sensing of divalent cations and AMP at the membrane surface. The ADP and the phosphorylation site (His277) are highlighted in space-filled representation. During the phosphor-transfer reaction, the phosphate group is translated from His277 to the PhoP aspartic acid (Asp51) (green arrow).

PhoQ forms a multidomain transmembrane homodimer, whose complete molecular structure is largely unresolved (Figure 1). Consequently, the mechanism underlying the PhoQ response to stimuli is not fully understood, and several mechanisms have been proposed to account for signaling transmission across the bacterial membrane, including piston shift, helix rotation and unwinding of a coiled-coil domain [6], [7]. A major obstacle to elucidating the signal transduction mechanism of the PhoQP TCS is the lack of a full atomistic structure of PhoQ. The following four different regions can be defined and appear to have distinct functions (Figure 1): (i) The periplasmic region consists of a sensor domain (SD) that detects changes in the periplasmic environment. Two opposing crystal structures have been solved (1YAX [8] and 3BQ8 [9]); Goldberg *et al.* [10] have recently showed that the most probable physiological arrangement is described by the 3BQ8 x-ray structure. This is consistent with disulfide scanning experiments, and can explain the topology and connectivity of the SD with the remaining domains of PhoQ. (ii) Two membrane-spanning antiparallel helices (called hereinafter TM1 and TM2) constitute the transmembrane (TM) region of PhoQ (Figure 1). (iii) Emerging from the cytoplasmic-facing membrane is a small signaling domain composed of a helix-loop-helix structure known as the HAMP region, and is thought to play an important role in transmitting the signal to the catalytic domain [11]. Finally, (iv) the catalytic or histidine kinase (HK) domain regulates the phosphoryl-state, and ultimately leads to PhoQ's function mainly as a kinase, or phosphatase during PhoP dephosphorylation. The phosphotransfer cycle between PhoQ and PhoP ensures, therefore, a robust and efficient genetic switch [12] (Figure 1).

In this study, we focus on the structural characterization of the transmembrane portion of PhoQ from *E. coli*. Difficulties in solving high-resolution x-ray structures of properly folded membrane proteins continue to be a significant barrier to determining structures at the atomic level and thus alternative, lower-resolution modeling strategies are often used as a proxy to obtain structural and functional information for membrane proteins [13]–[17]. Such strategies combined with computational methods can produce near-atomistic models; for example, replica exchange molecular dynamics (MD) was used to generate structural models for YycG sensor kinase consistent with mutagenesis studies [18]. The PhoQ protein might be a particularly good target for MD simulations since they are intrinsically well suited for studying the structures that are in dynamic equilibrium. When PhoQ activity is measured using transcriptional reporters, there is only a 2.5 to 3.5-fold difference in its activity in saturating Mg^2+^ concentration with respect to the basal state. This suggests a very small energetic difference in the two states, in the order of a single kcal/mol, assuming that the level of transcription reflects the fraction of the protein in the kinase mode. It is likely that multiple conformations observed by MD might be related to kinase or phosphatase activity. Thus, we used MD simulations to investigate the assembly of the PhoQ TM domain and the formation of a stable tetrameric bundle. MD simulations were guided and the resulting models validated by experimental cross-linking data. The TM domain connects with known, existing structures of the SD periplasmic domain as well as with the cytoplasmic HAMP domain. Therefore, the characterization of TM will provide insights into how conformational changes at the periplasmic SD might be transmitted to the cytoplasmic HAMP.

In order to test the validity of the models, we also simulated point mutations involving the polar residue Asn202 [19], which appears to be relevant for the solvation and signaling function of the TM domain. PhoQ exists in equilibrium between phosphatase-active and kinase-active conformations, and mutations of this residue strongly influence the signaling ability of the protein. This likely supports a water-filled pocket, as also seen in TM domains of another HK structure, HTRII from bacteriorhodopsin [20]. We found that Asn202 is indeed crucial for the hydration of the PhoQ TM bundle. Importantly, this feature was found to directly trigger a combined scissoring movement and ∼20 degree rotation of the TM helices that is propagated to the cytoplasmic side. This reveals the key role of the TM region in transmitting and modulating the signal through the bacterial inner membrane.

## Results

### The tetrameric assembly of the TM domain reveals an Asn202 inter-helical lock

Recently, disulfide cross-linking scanning was used to determine pairs of residues in close proximity in the PhoQ sensor domain [10]. The quantification of intermolecular disulfide formation showed a distinct periodic arrangement, fluctuating from almost 100% at peak efficiency to nearly zero at the lowest points, and guiding the modeling of the most biologically relevant sensor domain arrangement. The topology of the PhoQ homodimer indicates that four segments span the bacterial membrane (Figure 1). Therefore, by using the same procedure as for the sensor domain, disulfide cross-linking scanning experiments were performed for the TM region. Maxima occurred at residue positions 32, 35–36, 39–40 in TM1 and 199, 201–202, 205 in TM2, corresponding to the outer two-thirds of the TM domains (Figure S1 and Text S1). No significant cross-linking was observed near the cytoplasmic end of TM1, and the cross-linking was weak at this region of TM2, before gaining in intensity as the helix transitioned from the membrane and entered the cytoplasmic HAMP domain. The α-helical periodicity of the cross-linking results strongly suggested that the TM domain would assemble as a helical bundle, with the cross-linked residues facing towards the bundle core. These initial results along with available structural arrangements of domains connected to the TM (*i.e.* HAMP and sensor domain) strongly suggest a tetrameric helix assembly of the TM domain. We therefore modeled the TM1 and TM2 segments as ideal α-helices, and investigated their mutual interactions *ab initio* using MD simulations of the protein embedded in a lipid membrane bilayer; the disulfide cross-linking data was then used to validate the resulting models.

**Figure 2:**
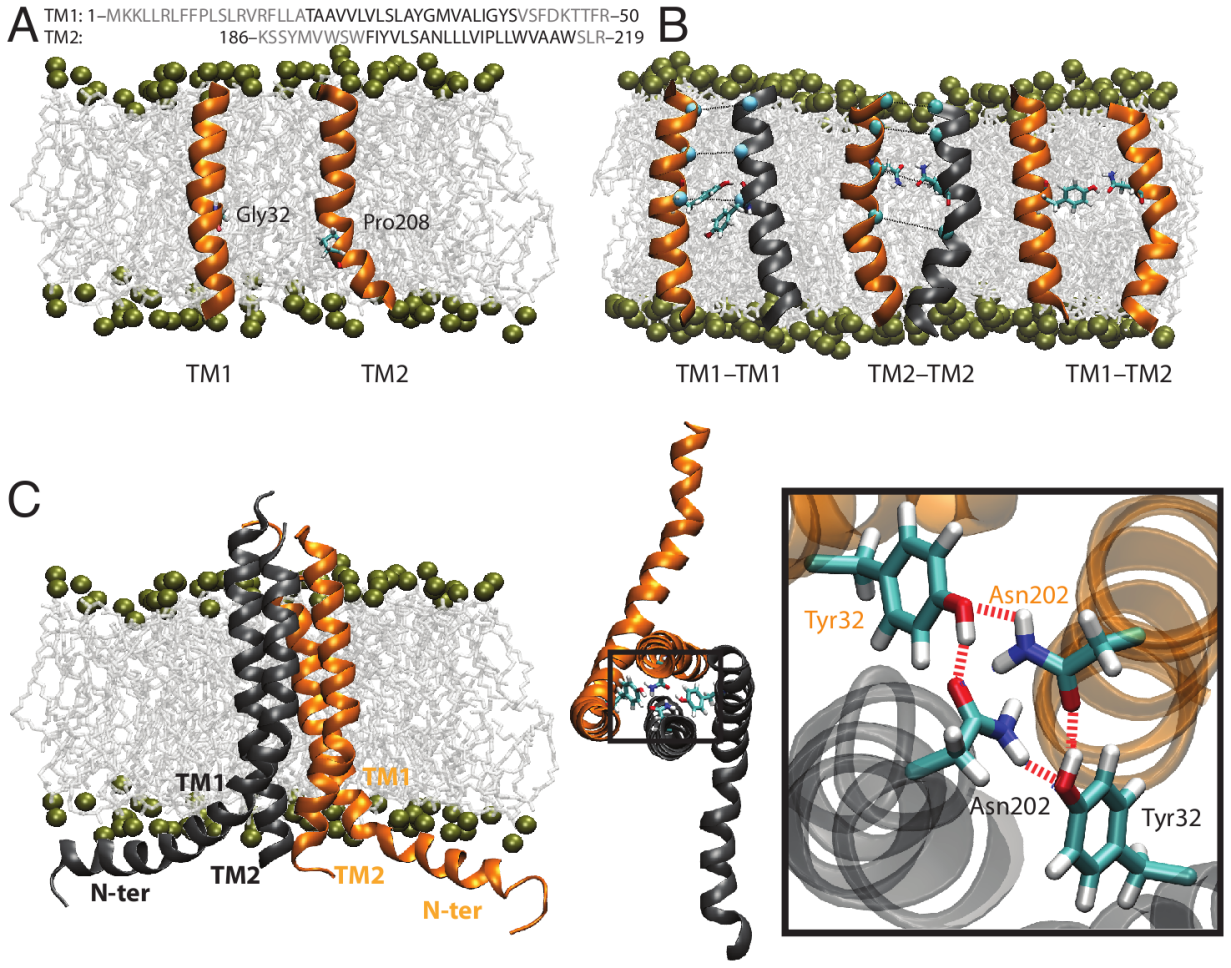
Assembly of the TM domain. (A) Models of individual TM1 and TM2 helices equilibrated in an all-atom membrane bilayer. Gly32 and Pro208 produce a kink in TM1 and TM2, respectively. (B) Stable TM dimers as obtained from MD in a united-atom membrane bilayer. (C) Tetrameric TM model structure produced using TM dimers, MD simulations and cross-linking spatial restraints. From left to right, the model is presented embedded in the membrane from the periplasmic top view, and with a focus on the H-bond network formed by residues Tyr32 and Asn202, which contributes to stabilize the bundle.

MD-refined models for TM1 (Thr21 to Val42) and TM2 (Phe195 to Trp215) showed only marginal fluctuations of the secondary structure (population RMSD = 1.4±0.3 Å) (Figure 2A). This supports the predicted initial helical conformation and matches with the membrane hydrophobic core. The presence of a glycine (Gly33) in TM1 and a proline (Pro208) in TM2 are important characteristics of these helices. In fact, proline residues are known to induce distortions as large as 25 degrees with respect to the direction of the helix axis [21]–[24]; glycine may also create milder kinks in the helices [25], [26]. Pro208 formed a kink in TM2 (28±7 deg) as did Gly33 in TM1, but to a smaller extent (19±5 deg) (Figure 2A). We expect that both kinks might favor the presence of a coiled-coil-like helical assembly for the tetrameric TM1-TM2 complex. These equilibrated helices were used as initial atomistic models to study the assembly of the TM complex.

The homo- and hetero-dimer assembly of TM1 and TM2 helices were analyzed with MD simulations. In this case, the predictions of MD simulations can be directly compared to available disulfide scanning experiments, that have probed the mutual interactions of the TM1-TM1′ and TM2-TM2′ homo-dimers, and to a smaller extent the TM1-TM2 hetero-dimers, within the PhoQ TM complex. This set of simulations was conducted with a high concentration of TM segments in a united-atom lipid bilayer to increase the conformational sampling (see Experimental procedures section). TM1 helices showed a strong tendency to form stable homo-dimers (60% of the total population, RMSD 2.1±0.4 Å). In all TM1 dimers, hydrophilic residues (Thr21, Ser29, Tyr32, Tyr40) face the dimer interface (Figure 2B). Self-assembly of TM2 helices also produced a stable cluster of dimers (RMSD 2.7±0.7 Å). The distortion caused by Pro208 led to a coiled coil-like assembly for TM2 dimers (Figure 2B). Asn202 plays a central role for the stability of the TM2 dimer. The Asn202 pair consistently forms two hydrogen bonds between their side chains. If only a single hydrogen bond is formed, then the dimer disassembles during the simulation. As previously reported [19], Asn202 is highly conserved in the transmembrane domain of TCS's and our models point to a structural role during dimer assembly. Moreover, experimental data indicate that the PhoQ function is impaired when Asn202 is mutated (e.g. during cross-linking), in agreement with this model, thus indicating a functional role of this residue in signaling conduction.

We also used experimental cross-linking data to validate the helix-helix interface of the dimer models produced by MD simulations. All residues showing high cross-linking efficiencies for TM1 and TM2 (TM1: Tyr32, Val35 Ala36, Tyr40; TM2: Leu199, Ala201 Asn202, Leu205) are in close proximity in the dimer models (Figure S2), thus providing a solid basis for further investigating the tetrameric assembly. We also probed the interactions along the TM1-TM2 interface: TM1 and TM2 domains associate in the membrane, forming oligomers. Two-thirds of the oligomers assemble in a conformation where Tyr32 interacts with Asn202 (Figure 2B). Moreover, these structures were in agreement with the cross-linking we were able to observe for the TM1-TM2 interface, where Ile207 was in close proximity to Val25 and Leu26 (Figure S3).

**Figure 3:**
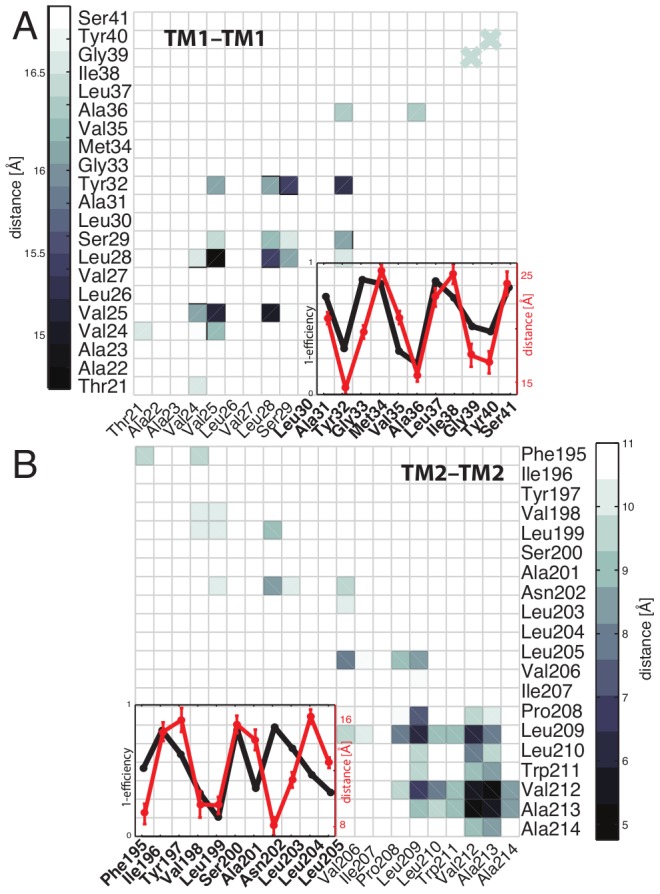
TM structural validation using disulfide cross-linking scanning. MD-averaged contact maps for (A) TM1 and (B) TM2 interfaces within the assembled TM domain. A direct comparison with cross-linking efficiency of (A) TM1 and (B) TM2 is reported in the inset, and shows a strong correlation between the cross-linking (1-efficiency) (in black) and the MD-averaged Cα distance measured for the TM model structure (in red). The cross-linking efficiency for the whole TM1 and TM2 regions is reported in Figure S1.

The TM1/TM2 homo/hetero-dimer structures were used to form a tetrameric conformation, and available cross-linking efficiency data were converted into semi-harmonic spatial restraints and applied during the first 10 ns of MD to equilibrate several initial tetrameric conditions. MD simulations were carried out afterward without any applied restraints on the bundle and converged to a stable conformation for the TM domain during ∼80 ns of MD. The final structural ensemble of the TM domain is characterized by an approximate 2-fold symmetry (Figure 2C); TM2 helices are more tightly packed than to TM1 and this is consistent with the overall stronger cross-linking efficiency reported for TM2 (Figure S1). The coiled-coil TM1 and TM2 dimer is within a 1-Å RMSD of a Crick-ideal backbone [27], [28]. All polar residues of TM1 (e.g. Thr21, Ser29, Tyr32, Tyr40) face the interface of the assembly. This final TM assembly is compatible with experimental cross-linking data, as shown by the correlation between the cross-linking efficiency and C_α_ contact maps calculated from MD simulations (Figure 3). The periodicity remains in close agreement with the predicted interface from cysteine-cross-linking mutagenesis. The predominant bundle conformation is stabilized by extensive van der Waals interactions throughout the bundle. Additionally, hydrogen-bonded interactions involving Asn202 and Tyr32 may contribute to stability. These polar amino acids formed a hydrogen-bonded network in many conformations, mainly involving the hydroxyl group from Tyr32 and the amide of Asn202 (Figure 2C). Previous studies showed that Tyr32 is not essential for signaling, and that various residues of different size, shape and hydrogen-bond donors and acceptors complementarity can replace Asn202 [19]. Thus, the specific hydrogen-bond network seen in the model may not be required for attaining the kinase-active conformation. Instead, it could be representative of structures formed in the resting phosphatase-active state.

### Connections between the TM bundle and neighboring domains

The minimal TM structure was extended to help model the connections to the domains outside the lipid bilayer surface. N-terminus to TM1 is a 20-residue sequence with a high potential to form a surface-seeking amphiphilic helix [29], hereafter referred to as the cytoplasmic N-terminal amphiphilic helix (Figure 2C). At the C-terminus of TM1 we also included residues 43–50, corresponding to a portion of the helix that forms the dimer interface of the neighboring periplasmic sensor domain. We also extended the TM2 helix from its C-terminus to include its connection to the HAMP domain. The additional segments were appended in a helical conformation to the TM structure, inserted in an all-atom membrane, and simulated over 150 ns of MD (population RMSD = 1.9±0.3 Å). The TM bundle was stable during this timescale and maintained the same core interactions as described above (Figure 2C).

The N-terminal cytoplasmic helix folded onto the membrane in a surface orientation, as expected. The presence of a proline at position 10 induced a kink that bent the helix back making it parallel to the membrane surface in a rivet-like conformation (Figure 2C). This arrangement is also consistent with the amphipathic nature of the Met1 – Pro10 segment, and might be required for the anchoring and signal transduction through the membrane. The distance between TM2 C-termini is consistent with the NMR [30] and X-ray [31] structures of HAMP, and the periplasmic side of TM1 is well positioned to connect to the SD domain (namely pdb 3BQ8 [8]).

### Asn202 is crucial for the solvation of the TM domain

Recently, Goldberg *et al.* [19] showed that Asn202 in TM2 is critical for signal transduction. When Asn202 is mutated to non-polar residues, the transcription of PhoPQ-regulated genes is impaired. Thus, it was proposed that the polarity of Asn202 and the possibility to accommodate a water pocket at the TM core could potentially play an important role for kinase activity and signal transduction. Using the assembled model for TM, we further investigated the solvation of the bundle and monitored the structural determinants of Asn202 and other polar residues within the bundle (i.e. Tyr40, Ser43, Lys46, Thr47, Arg50, Lys186, Ser193, Tyr197, Ser200). Their arrangement forms a “hydrophilic ladder”, and allows water molecules to diffuse into the membrane only from the periplasmic side. They progress discretely from residue-to-residue, by either interacting with the hydrophilic side chains or with the backbone. During MD, we observed transient solvation of the TM bundle. Lys46, Arg50 and Lys186, due to their long aliphatic chain and polar head, enhance water permeation in the membrane. Water molecules then access the membrane's hydrophilic core by interacting with Ser193, Thr47 or Ser43, before finally entering the bundle's center at the Asn202 position (Figure S4).

**Figure 4:**
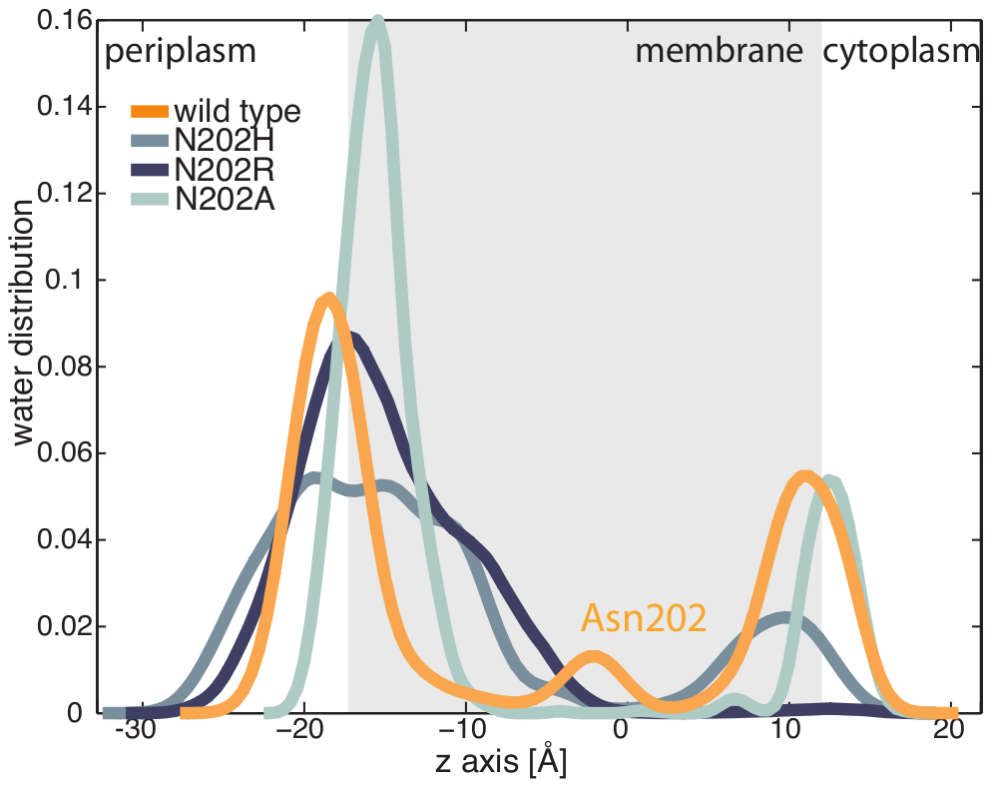
Effects of Asn202 mutation on the solvation of the TM domain. The kernel density estimation of water molecules for MD simulations of the wild-type TM bundle, and three relevant Asn202 mutants: N202A, N202H, and H202R. Residue 202 is localized in the middle of the membrane (at 0 Å). Conservative mutations preserve the hydration of the TM core, while substitution with alanine prevents water to enter the bundle. Distribution is calculated along the axis orthogonal to the membrane bilayer, and the transmembrane portion is schematically indicated by the grey area defined by the MD-averaged distance between bilayer polar heads (namely, phosphorus atoms).

The presence of water in the middle of the bundle is consistent with the water-containing cavity hypothesis [19]. We introduced selected mutations in our model, namely N202A, N202R and N202H, to test their effect on the solvation of the bundle. *In vitro* experiments showed that N202A mutation fully impaired the kinase function of PhoQ. In MD, N202A resulted in an important rearrangement of the tetramer hydrogen bond network. The hydrogen bond network, observed for the wild-type TM bundle, is totally disrupted, and Tyr32 formed hydrogen bonds with the backbone of Ala202. Furthermore, the mutation prevented the water molecules from completely entering the bundle (Figure 4). *In vitro* experiments involving N202R and N202H showed increased kinase activity compared to the wild-type [19]. During the simulation, the N202R mutation, which was also the most “activating” mutation, had the greatest effect on hydration and structure. The Arg202 side chain stretched towards the periplasmic side of the membrane interface and attracted water molecules into the bundle (Figure 4). The core of the TM bundle remained solvated throughout the entire simulation. The snorkeling of Arg202 prevents Tyr32 from engaging in the same interactions as in the wild-type. The N202H mutant preserved the H-bond network: A single water molecule remained trapped in the hydrophilic cavity throughout the entire simulation (Figure 4). Our analysis seems to indicate that His202 is more efficient in keeping the cavity solvated. When the center of the bundle is solvated, TM1 bends towards the center of the tetramer for both mutations, reducing the inter-helical distance to approximately 17 Å. Thus, the electrostatic features and the general structure of our TM model allow for the solvation of the bundle. This fully supports the water-cavity hypothesis previously proposed and points to the crucial role of water for easing the mechanism involved in signal transmission defined by TM1 and TM2 segments (see following sections).

### Solvation triggers the conformational rearrangement of the TM domain

**Figure 5:**
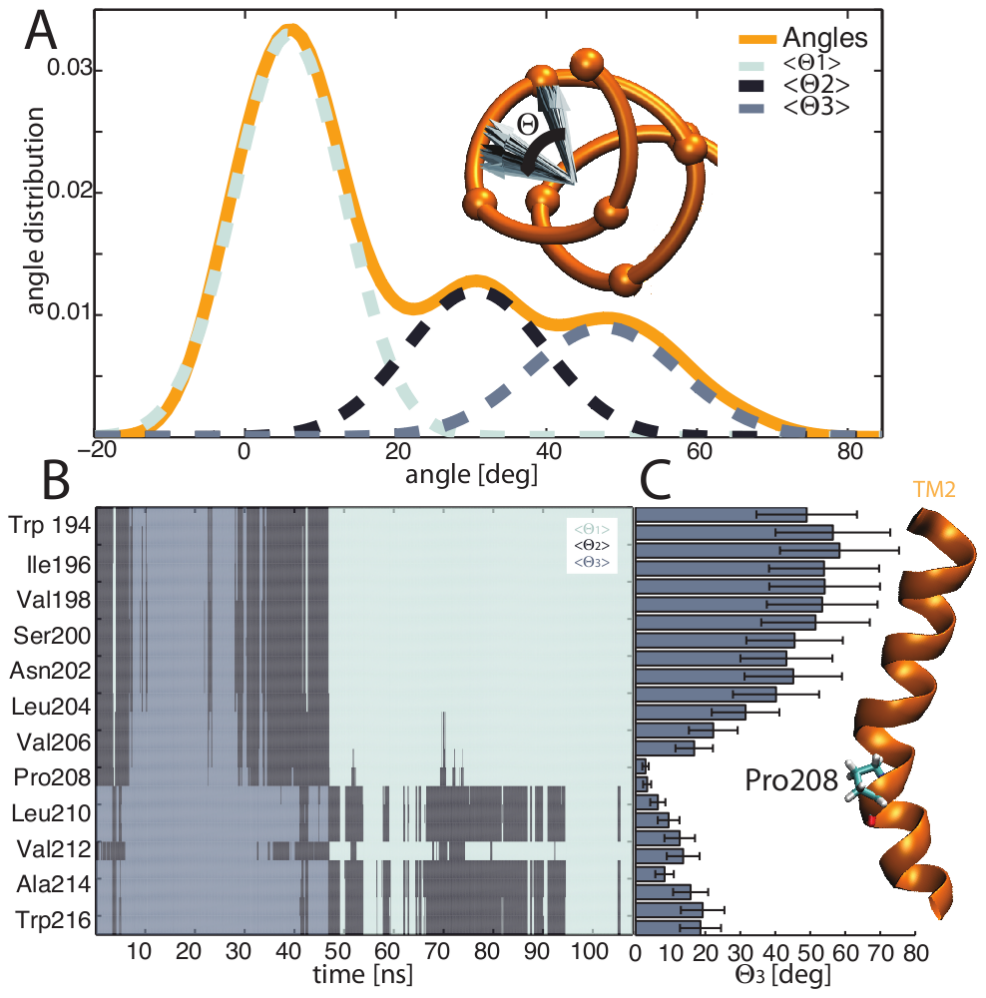
Solvation-dependent dynamic features of the TM domain. (A) The rotation of the Cα atoms around the TM2 helix main axis is computed based on a principal component analysis, and the angle distribution is characterized by three major modes, that can be fitted using three Gaussians, (B) MD time series of the TM2 residue state corresponding to the angle distribution. The system is initially in a metastable state (black), before switching to a solvated state where the TM1-TM1 interface is tighter (grey). After ∼30 ns, the system, passing through to a metastable state, shifts to a stable state characterized by a larger TM1-TM1 distance (light grey). (C) The rotation per residue related to the switch between the 2 most relevant states in MD (B) is calculated. TM2 Pro208 acts like a hinge, and transforms the large movement of the N-terminus into a mild rotation (∼20 degrees) of the TM2 residue at the cytoplasmic interface.

The transition between a solvated and non-solvated state observed in MD is associated with a conformational change in the wild-type bundle, i.e. a scissoring movement at the periplasmic surface of the membrane characterized by TM1 helices bending towards the center of the bundle, thus causing TM2 to spread apart. This scissoring movement, however, does not seem to propagate fully to the TM2 C-termini. To further characterize this conformational change, a principal component analysis (PCA) was performed based on the positions of the C_α_ atoms during the MD simulation. PCA has been shown to be an effective tool to remove thermal noise and retrieve significant movements of a system [32]. The main principal components were computed and used as a new coordinate system for the projection of the position of the TM bundle's C_α_ atoms. The rotation around the helix main axis was then extracted for TM2 (Figure 5A). The corresponding angle distribution has three modes that can be described using three Gaussians (Figure 5A). Three centroids were defined by using the k-means clustering method, each representing a distinct state. In each frame of the simulation, the position of the C_α_ was classified in one of the three states. When plotted against time, each cluster corresponds to a well-defined conformational state in the simulation (Figure 5B). During the transition state, a minor (∼10 deg) rotation of TM1 C-termini allows water molecules to enter the membrane and eventually solvate the center of the bundle. The solvation of this cavity triggers the previously described conformational change. The scissoring movement of TM1 imposes a rotation of TM2 that is propagated to its C-terminus. It is interesting to note that Pro208 acts like a hinge, transforming a large TM2 rotation (>45 degrees) coupled to a scissoring movement (6 Å) at the periplasmic side into a pure TM2 rotation at the cytosolic side (Figure 5C). Cross-linking experiments showed that even though PhoQ was still functional, mutating Pro208 decreased the activity of PhoQ. This is consistent with sequence analysis using BLAST [33], [34], where we found that proline at position 208 is highly conserved, thus supporting its functional role. The final rotation of TM2 C-termini is approximately 20 degrees. The helical conformation of the TM region also induces a minor coupled piston-like translation of TM2 (0.8±0.1 Å). This could influence the stability of HAMP and trigger the signal transduction [35]. Furthermore, the amplitude of the piston movement is in agreement with the experimental observation of the nitrate-sensing protein (NarX) structure [36]. This complex dynamic rearrangement was not observed for the N202A mutant that consistently remained locked in a rigid conformation with the lack of solvation of the bundle. Instead, the activating mutations (N202H and N202R) were able to explore the wild-type conformation characterized by a large solvation of the bundle, and are expected to readily adapt to conformational changes of the periplasmic SD to modulate the HAMP domain during PhoQ kinase activated state.

### A coupled TM movement provides insight into the signal transduction mechanism

**Figure 6:**
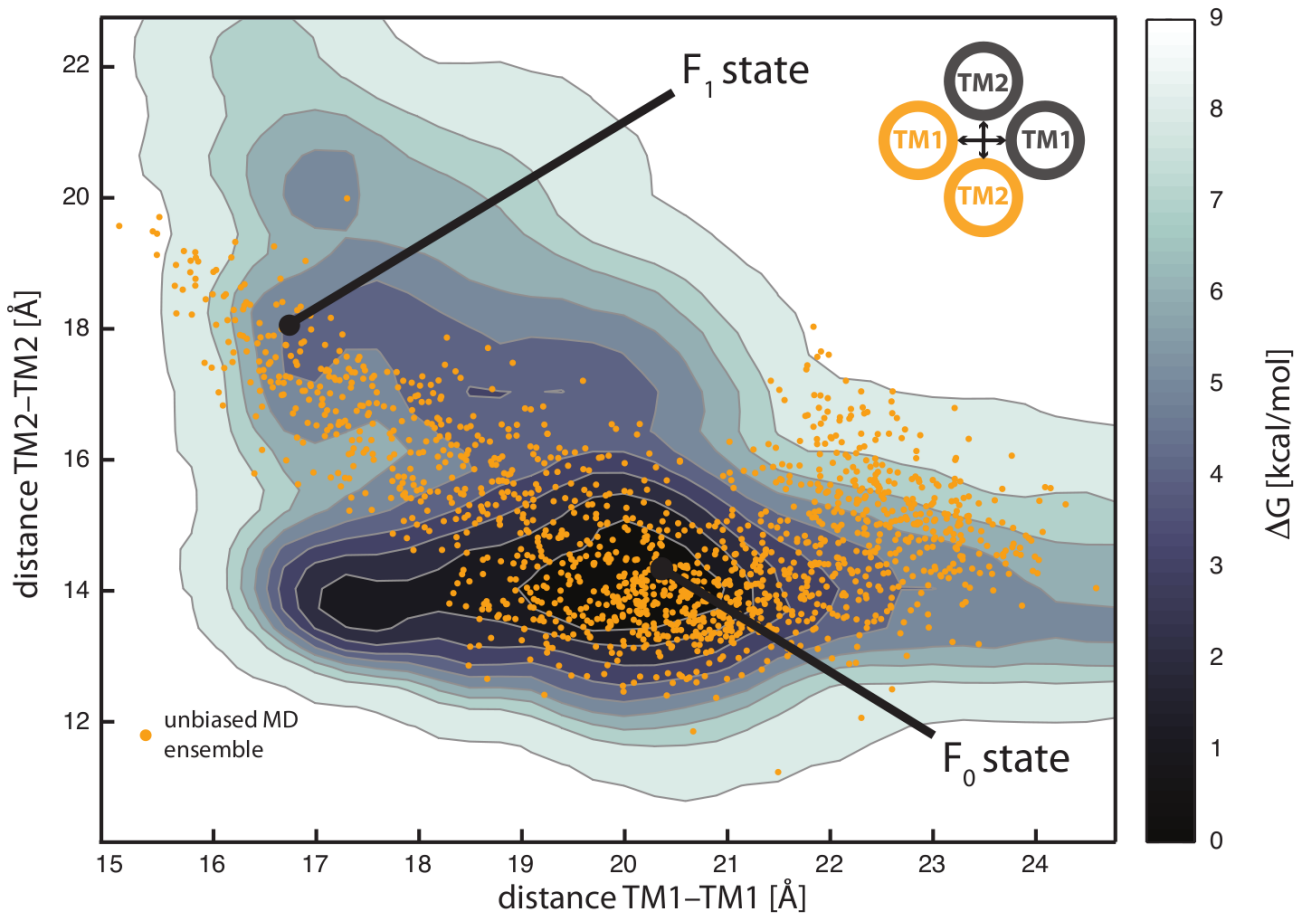
Free energy landscape for the TM conformational change. The free energy landscape defined by sampling inter-helical distances between TM1 C-termini and TM2 N-termini is reported. The conformational change observed in the unbiased MD simulations (orange points) occurs along a free energy valley, that connects a main equilibrium state (*F_0_*) and a high-energy conformation, and can be associated with relevant states during the signaling process (*F_1_*, ∼5 kcal/mol higher in free energy).

Unrestrained MD provided us with useful information about solvation and atomistic features of the bundle, including a possible coupled movement of TM1 and TM2. However, we were unable to exhaustively sample the large conformational space associated with TM dynamics, especially for higher energy states that could be relevant for the different kinase/phosphatase states of PhoQ. For this reason, we used enhanced sampling techniques to explore the dynamic determinants of the TM domain. Among those methods, metadynamics has been shown to be a valuable tool in simulating rare events and reconstructing the free energy landscape of biomolecules [37]. In order to characterize the free energy landscape associated with TM conformational changes, we used the distances between TM1 C-termini and TM2 N-termini as main collective variables. They appear to describe the major large-scale fluctuation of the TM domain in free MD. They allowed the sampling of the opening and closing movements taking place at the periplasmic side of the membrane. Metadynamics confirmed the equilibrium configuration ensemble (F_0_) found in free MD characterized by an inter-helical distance of ∼20 Å for TM1 and ∼14 Å for TM2 (Figure 6). It is interesting to note that the free energy landscape associated with TM1 and TM2 movement showed a broad valley (∼5 kcal/mol higher with respect to the F_0_ equilibrium conformations), that extends to shorter TM1 (∼16 Å) and larger TM2 (∼19 Å) distances, thus capturing a second alternative conformation state (F_1_), already transiently sampled during free MD simulations. Moreover, as in previous unbiased MD simulations, the conformational change along the valley is coupled with a ∼20 degrees rotation of TM2 C-termini. This slightly higher energy region found in multiple metadynamics runs is probably representative of conformations sampled by TM during signal propagation. In fact, given the small energy difference between the two states, which might be comparable or lower than the effect of changing the phospholipids composition of the bacterial membrane (event that is likely to happen during sensing of cationic species at the perisplasmic side), we assume that both states might be representative of equilibrium-like configurations of either the kinase-active or phosphatase-active states.

## Discussion

Currently, no soluble NMR or X-ray crystal structure has been solved for the PhoQ transmembrane domain, thus hindering a thorough understanding of the signal transduction mechanism from the periplasmic sensor to the cytoplasmic kinase domain. In this study, we have combined all-atom molecular dynamics simulations with experimental cross-linking disulfide scanning data to build a four helical bundle model for the *E. coli* PhoQ TM region. Experimental cross-linking data provided a set of low-resolution spatial restraints for TM packing, thus validating a structural ensemble of the TM domain generated by MD simulations. The accuracy of the proposed structural ensemble might be limited by the absence of the adjacent PhoQ domains during the modeling and by the current sampling limitations of MD methods. However, the ensemble appears to recapitulate much of the experimental data observed for PhoQ. As discussed in more detail below, we see agreement with the experiments and find insights into the signaling of histidine kinases. First, the simulations provide an ensemble of molecular models that are consistent with the disulfide cross-linking data, particularly over the regions where strong cross-linking is observed. Secondly, they demonstrate the essential role of Pro208, which causes a bend in the TM2 helix. This bending provides a mechanism to modulate the amplitude and combination of motions in the TM domain. Finally, simulations of several mutants are compared to the experimental activity measurements, leading to a correlation between the degree of hydration of the bundle's core and the conformational changes of the TM domain.

Cross-linking data support a four-helix bundle for the PhoQ transmembrane domain (Figure S1). During MD, the packing of the side chains formed a stable coiled-coil tetrameric arrangement (within 1 Å RMSD of a Crick-ideal backbone), characterized by an approximate 2-fold symmetry. Moreover, we identify an N-terminal cytoplasmic amphiphilic helix lying along the membrane surface. This could help anchor TM to a specific location in the membrane bilayer and in turn, may favor the signaling transmission. Furthermore, the tetramer assembly is fully consistent with disulfide scanning data. The registration and packing of the helices in this structure is fully compatible with our model (RMSD 2.1 Å), and the structural difference between the two models is consistent with the resolution that is characteristic of the approach used to model the TM region. Although some aspects of the disulfide cross-linking studies are well explained by the model, others would appear more difficult to rationalize. For example, experimentally one observes very low cross-linking efficiency for TM1 and TM2 at the cytoplasmic interface (Figure S1). On one hand, the tight packing of this region, as observed in our model, could preclude the diffusion of reagents needed to induce disulfide formation. On the other hand, the diagonal helices of the TM bundle might also explore more distant conformations during signaling, as observed in the related TM bundle of HtrII, where the helices are under-packed in this region of the structure [20].

**Figure 7:**
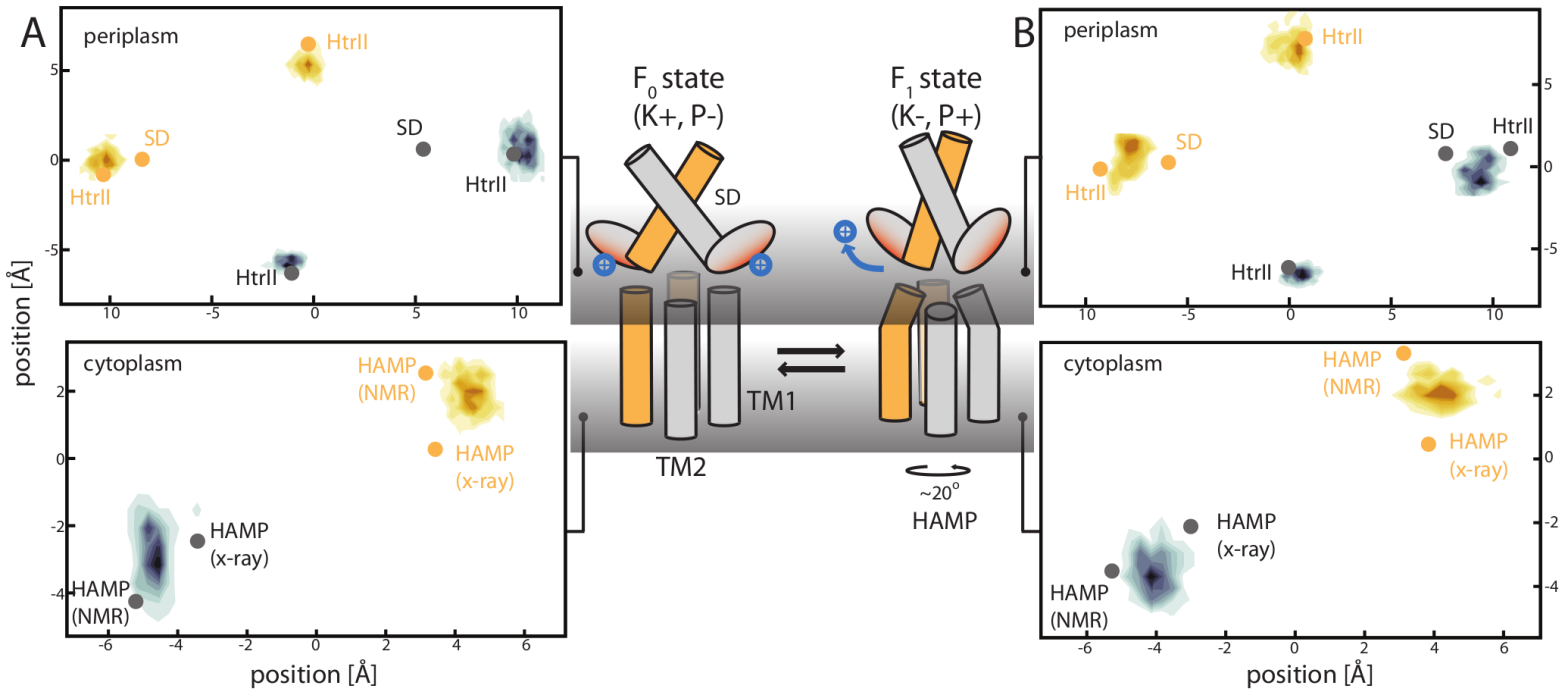
TM connectivity and implications for signaling mechanism. The panels (A) and (B) indicate the projection for the F_0_ and F_1_ states, respectively, on the periplasmic (top) and cytoplasmic (bottom) surface of the membrane of the helical termini of the TM model. The contour lines represent the position of TM1 and TM2 during MD, and the dots represent the position of SD and HAMP available structures at the same section surface. (Central panel) At high mM concentration of Mg^2+^ and Ca^2+^ cations, metal bridges are formed between the SD acidic cluster and the negatively charged membrane. This conformation can be associated to the F_0_ state of our model and to a kinase-dominant state (K^+^). When the concentration decreases, the metal ion bridges are disrupted, leading to repulsion between the membrane and SD active site. This triggers a conformational change of the TM domain (F_1_ state associated to a phosphatase-dominant conformation, P^+^), and results in a rotation of TM2 at the cytoplasmic interface, that is then transmitted to the linked HAMP domain.

Despite this discrepancy, the topology of the bundle is consistent with the available structures of the periplasmic (SD) and cytoplasmic (HAMP) domains. At the periplasmic membrane surface, when the bundle is solvated, TM1 helices can easily connect to the biologically-validated dimeric conformation of the sensor domain (pdb: 3BQ8 [9]) (Figure 7). On the cytoplasmic surface of the membrane, the TM2 C-terminal helices can be extended to merge with the two currently available structures of the HAMP domain (Figure 7). Furthermore, we observed that the kink induced by Pro10 at the PhoQ N-terminal amphiphilic helix is functional, thus helping to avoid steric clashes with the helical structure of the HAMP domain (Figure 3).

MD simulations of this structural ensemble showed that the polar nature of Asn202 is crucial for the assembly and proper solvation of the TM domain. In the proposed model, Asn202 forms a hydrogen-bonded network with water and other residues in the lumen of the TM region. Furthermore, the proximity of other polar residues at the periplasmic end allows water molecules to enter the bundle and reach its core. We showed that the removal of this polar residue in the N202A mutant (for which *in vitro* the kinase activity is completely impaired [19]) prevents the solvation of the TM core, thus locking the domain into a rigid one-state conformation. Mutations to histidine or arginine partially preserve the electrostatic nature of the native Asn202. This produces a similar hydration of the TM core and indicates a more flexible conformation of the TM domain. Consistently, the kinase activity of these mutants is conserved and is even higher than the wild-type [19]. Therefore, the degree of solvation of the TM domain appears to be strongly correlated with the kinase activity of PhoQ. This points to the key role of polar residues located within the bundle and water molecules in facilitating signaling through the membrane.

It is interesting to see how this model can help elucidate the mechanism of signaling, and how its structure and dynamics relate to the SD and HAMP domains. The SD X-ray structure (3BQ8 [9]) can optimally be connected to the TM state observed during MD where the TM1 helices are closer together (F_1_ in Figure 6). Therefore, the F_0_ state, featuring the opening of TM1 helices, could be associated with the yet uncharacterized SD conformation. The stimuli (e.g. change in the concentration of cations) could drive the rearrangement of the SD dimer, causing it to pivot around its inter-helical dimeric interface (Figure 7). This scissoring movement would change the angle and inter-helical distance at which TM1 helices enter the membrane, thus promoting a concerted rearrangement of TM1 and TM2 helices, as observed in our simulations. Clearly, further studies are needed to unveil the dynamic features of the SD-TM coupling. Nonetheless, several experimental studies have shown that the TM helices undergo a structural rearrangement during signaling [38]–[40], and that a sensor domain-independent mechanism also exists [41]. Therefore, the complex interplay between scissoring and rotational movements at the periplasmic side of the membrane results, as revealed by our model, in a simple clockwise rotation of the TM2 helices at the cytoplasmic side (∼20 degrees, Figures 5 and 7). This is consistent with the cogwheel mechanism proposed for the HAMP domain [30] (Figure 7). Therefore, we conclude that the two relevant conformations observed in MD for the TM domain might be representative of the two main states of PhoQ, i.e. kinase and phosphatase active. And, the interconversion between these two states modulates the rotation of the cytoplasmic HAMP domain and, eventually, the phosphorylation and dephosphorylation ability of the histidine kinase domain (Figure 7).

Finally, this study shows how molecular simulations combined with low-resolution spatial restraints extracted from experimental cross-linking data can be used to investigate the structure and assembly of transmembrane proteins. Although full coarse-grained MD approaches can correctly assemble helix oligomers in membranes [42]–[44], we adopted here an atomistic representation to better describe the helical kinks and the important solvation effects at the PhoQ TM domain. This protocol is of general applicability and can easily be extended to study other transmembrane protein complexes. In this case, the characterization of the PhoQ TM domain, not only sheds new structural light on two-component system signal transduction across membranes, but also can be exploited for the design of specific drugs or peptidomimetics capable of impairing PhoQ assembly and function.

## Materials and Methods

### Structural models

No structural data exist for TM1 and TM2. Thus, we modeled their structures *ab initio*. We combined 8 topology prediction algorithms to isolate the transmembrane domain (TM) of PhoQ (Table S1). The transmembrane portion of TM1 was roughly identified from residue Thr21 to Val42, and TM2 from Phe195 to Trp215. Both transmembrane domains were modeled as ideal α-helices based on the combined results of secondary prediction tools such as HNN [45], Jpred [46], [47], NetSurfP [48], PSIpred [49], ProteinPredict [50]. These initial predictions were used to build the atomistic models of TM1 and TM2 embedded in the lipid bilayer. The MD simulations were then used to equilibrate them and to find the correct match with the membrane hydrophobic environment.

### Molecular dynamics simulations of the assembly

We used molecular dynamics (MD) simulations to further characterize the assembly of the TM domain. In a first step, the ideal helical TM models were each separately inserted and equilibrated in a 60×60 Å^2^ Palmitoyl Oleoly Phosphatidyl Choline (POPC) membrane patch [51] to characterize their isolated structure in a phospholipid bilayer. Then, the self-assembly of TM1 and TM2 was studied separately, producing a high-concentration of TM proteins in a 100×100 Å^2^ pre-equilibrated patch of united-atom dodecane membrane (DODE). The thickness of the DODE membrane is approximately equivalent the hydrophobic core of the POPC bilayer. Diffusion in an all-atom membrane model is slow and the protein oligomerization may require timescales not easily accessible within an all-atom MD. We inserted the equilibrated transmembrane domains TM1 and TM2 into a united-atom dodecane membrane (DODE) [52]. In a united-atom model, groups of atoms are clustered, thus decreasing the computation and diffusion time. In a second step, TM1 and TM2 dimer conformations compatible with cross-linking data were used to assemble a four-helix bundle. The resulting tetramer was inserted in a 55×55 Å^2^ DODE membrane bilayer. Since an important rearrangement of the side chains is expected, harmonic restraints were also added to Cα showing high cross-linking efficiency. Only TM2 dimers were restrained, due to the highly dynamic nature, i.e. Phe195, Val198, Asn202 and Val206 pairs were subjected to semi-harmonic restraints with force constant *k_f_* = 100 kcal/Å^2^ and the rest point *x_0_* to 12 Å. The harmonic restraints were removed after a 10 ns simulation. In addition, an extended version of the TM1-TM2 tetramer (TM1: Met1 – Arg50, TM2: LYS186 – ARG219), and three mutant species (N202A, N202H, N202R) were also tested in a 70×70 Å^2^ Palmitoyl Oleoly Phosphatidyl Ethanolamine (POPE) membrane.

All simulations were performed using NAMD [53] engine with the CHARMM27 force field [54], including CMAP corrections. We used metadynamics to explore the free energy landscape associated with the opening and closing of the TM domain at the perisplasmic side. In metadynamics, Gaussians are added to the energy surface and force the system to escape from local minima. This technique requires *a priori* knowledge of the degree of freedom relevant to a conformational change. In the current system, the observed conformational change was described by means of two collective variables representing the distance between the C-termini and N-termini of TM1 and TM2 respectively, and was the most relevant motion from a principal component analysis of the unbiased MD simulations. The inter-helical distances were defined using the center of mass of residue Ser43 to Thr48 for TM1 and Ser188 to Trp192 for TM2. A set of three metadynamics simulations (∼30 ns each) was performed using the collective variable module of NAMD. Gaussians of width 0.3 and weight 0.01 were inserted every 300 fs. The conformational space was sampled from 15 to 25 Å for TM1 and 10 to 20 Å for TM2 (Figure 6). The obtained free-energy profile was visualized as an iso-contour map with a grid spacing of 0.3 Å (Figure 6).

It should be mentioned that our models and simulations may be affected by several approximations: the assembly does not include the complete PhoQ homodimer, but only the TM domain and perturbations might be more evident at the protein termini; two collective variables may not be able to describe all the degrees of freedom of the system, leading to an approximation of the real energy landscape; furthermore, sampling can also be an issue to accurately describe solvation of the bundle. Therefore, multiple MD simulations were run from different initial conditions to ensure the reproducibility of the results. A list of all performed simulations is reported in Table S2.

### Crosslinking reactions and analysis

Covalent chemical cross-linking has shown to be a useful technique to probe protein-protein interactions and provide low-resolution structural information of the protein-protein reciprocal arrangement [55] [10], [15], [56]. If both cysteine residues are close to each other (<12 Å between two Cα), the cross-linking efficiency will be high. The spatial interaction between the different amino acids can then be deduced and provides spatial restraints on the folding and packing of the PhoQ TM region. In the current study using a procedure similar as in [10], we report cross-linking results for the TM1-TM1 and TM2-TM2 intermolecular interactions for the TM tetrameric bundle (Figures 3 and S1) [57]. We also report a limited set of data for the inter TM1-TM2 interactions centered on Ile207 at TM2 (Figure S3). To crosslink cysteine residues in the transmembrane domain we used the oxidative catalyst copper (II) 1,10 phenanthroline (Cu(II)Phenanthroline), a small membrane permeable reagent that efficiently catalyzes disulfide bond formation in the membrane. We treated a 10 µL sample of cell envelopes to 10 µL of buffer containing 2 mM or 0.2 mM Cu(II)Phenanthroline for a final concentration of either 1 mM or 0.1 mM. Reactions were allowed to proceed for 30 minutes at room temperature, and then stopped by the addition of 20 mM N-Ethyl Maleimide (NEM) and 20 mM EDTA. Reactions were spun at 16'000 rpm at 4 degrees C to concentrate membranes. Suspension in 8 M urea LDS loading buffer preceded gel loading. Western blotting was used to estimate cross-linking efficiency quantifying the fraction of cross-linked dimer to total visible protein (i.e. dimer/dimer+monomer, see details in Text S1 and Figures 3, S1, and S3).

## Supporting Information

Figure S1 **Fractional cross-linking of PhoQ TM residues.** The black curve represents the cross-linking efficiency of (A) TM1 and (B) TM2. The data can be fitted with a sine wave (dashed orange). N202 was excluded for the fit, since it is an inactivating mutation. The reported periodicity (ω = 3.62 and ω = 3.3 respectively) strongly suggested that the TM domain would assemble as a helical bundle. These data are used to validate our results for TM assembly and are compared with MD results in Figure 3 of the main text (see supplementary methods).
(TIFF)

Figure S2 **TM homodimer validation and selection via disulfide cross-linking scanning.** MD-averaged contact maps for (A) TM1 and (B) TM2 dimer interfaces. A direct comparison with cross-linking efficiency (Figure S1) of (A) TM1 and (B) TM2 is reported in the insets and shows a strong correlation between the cross-linking (1-efficiency) (in black) and the MD-averaged Cα distance measured for the TM model structure (in red).
(TIFF)

Figure S3 **Inter-TM crosslinking for Ile207.** Results of inter-TM crosslinking experiment between residue 207 on TM2 and a window of TM1 residues between 23 and 27. Error bars represent two independent experiments.
(TIFF)

Figure S4 **Water distribution around the TM bundle.** (Top) The water molecules in contact with the TM bundle for wild type (WT), and three mutants: N202H, N202R and N202A are displayed with red spheres (see also Figure 4 in the main text). (Bottom) The focus on the N-terminal helix extended from TM1 illustrates its amphiphilic nature in our model. The hydrophobic residues are displayed in white, the polar and positively charged residue in green and blue, respectively.
(TIFF)

Table S1 **Transmembrane domain predictions.** The transmembrane domain considered for the modeling is highlighted in grey as a consensus obtained from the following programs: SPOCTOPUS [59], OCTOPUS [60], PSIpred [49], TMHMM [61], HMMTOP [62], TMpred [63], topcons single [64], and DAS [65].
(PDF)

Table S2 **List of performed simulations.**
(PDF)

Text S1 **Supporting methods and materials.** Complementary details on the molecular dynamics simulation and cross-linking experiments.
(PDF)
