# Supplementary figures and images for: Assembly of the Transmembrane Domain of E. coli PhoQ Histidine Kinase: Implications for Signal Transduction from Molecular Simulations
Source: PLoS Comput Biol. 2013 Jan 24;9(1):e1002878. doi: 10.1371/journal.pcbi.1002878 (PMC3554529; doi:10.1371/journal.pcbi.1002878)

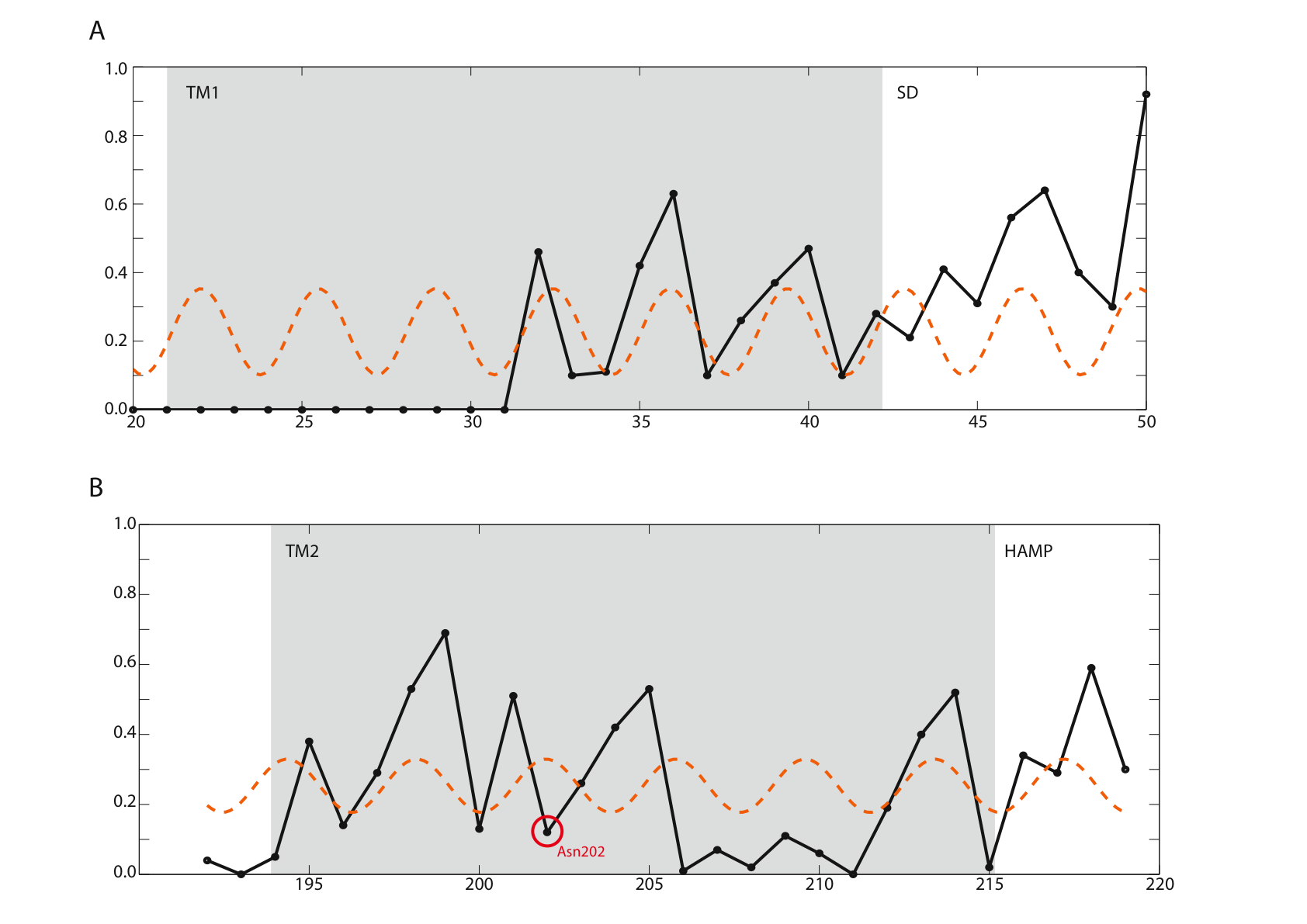

Supplement: Figure S1 — Fractional cross-linking of PhoQ TM residues. The black curve represents the cross-linking efficiency of (A) TM1 and (B) TM2. The data can be fitted with a sine wave (dashed orange). N202 was excluded for the fit, since it is an inactivating mutation. The reported periodicity (ω = 3.62 and ω = 3.3 respectively) strongly suggested that the TM domain would assemble as a helical bundle. These data are used to validate our results for TM assembly and are compared with MD results in Figure 3 of the main text (see supplementary methods). (TIFF) [file pcbi.1002878.s001.tiff]

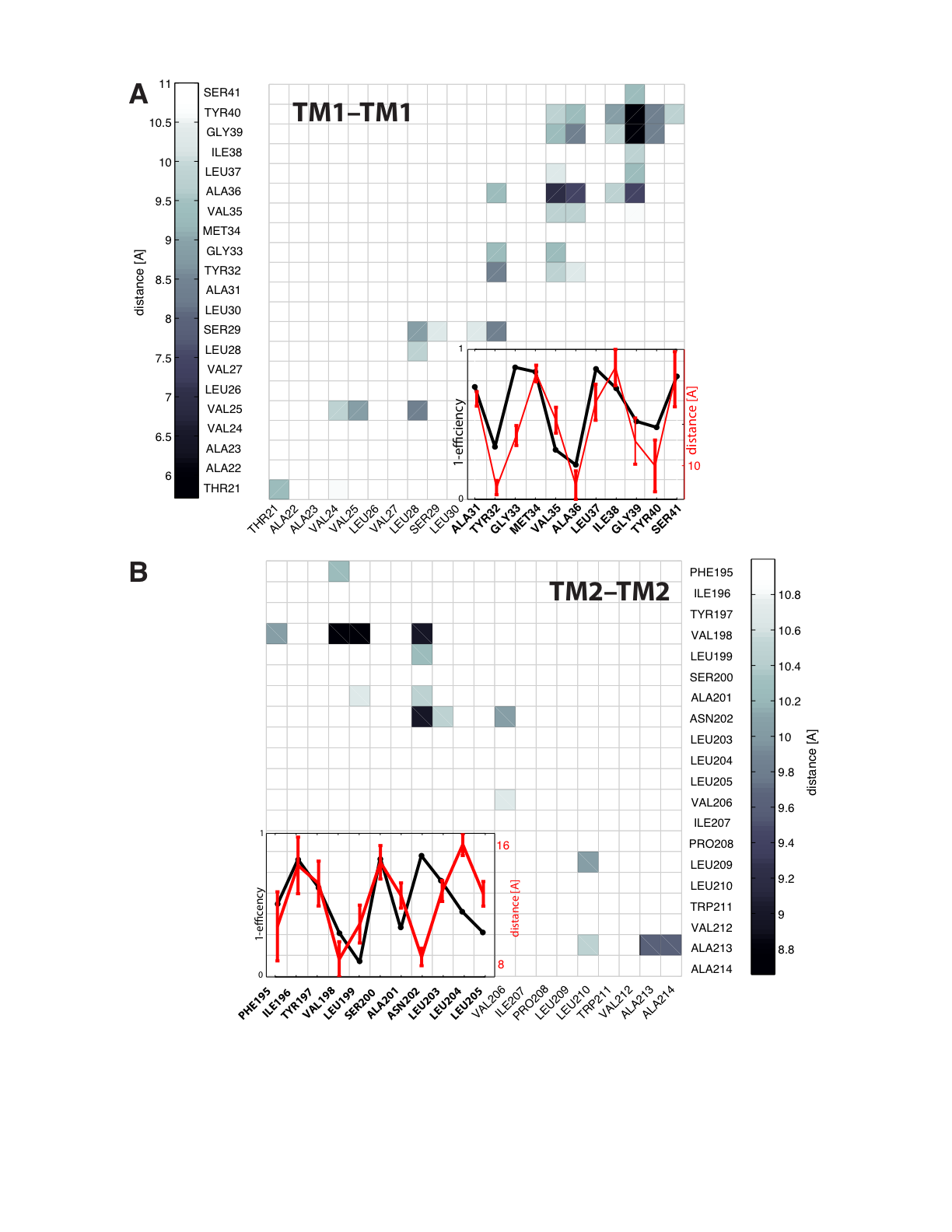

Supplement: Figure S2 — TM homodimer validation and selection via disulfide cross-linking scanning. MD-averaged contact maps for (A) TM1 and (B) TM2 dimer interfaces. A direct comparison with cross-linking efficiency (Figure S1) of (A) TM1 and (B) TM2 is reported in the insets and shows a strong correlation between the cross-linking (1-efficiency) (in black) and the MD-averaged Cα distance measured for the TM model structure (in red). (TIFF) [file pcbi.1002878.s002.tiff]

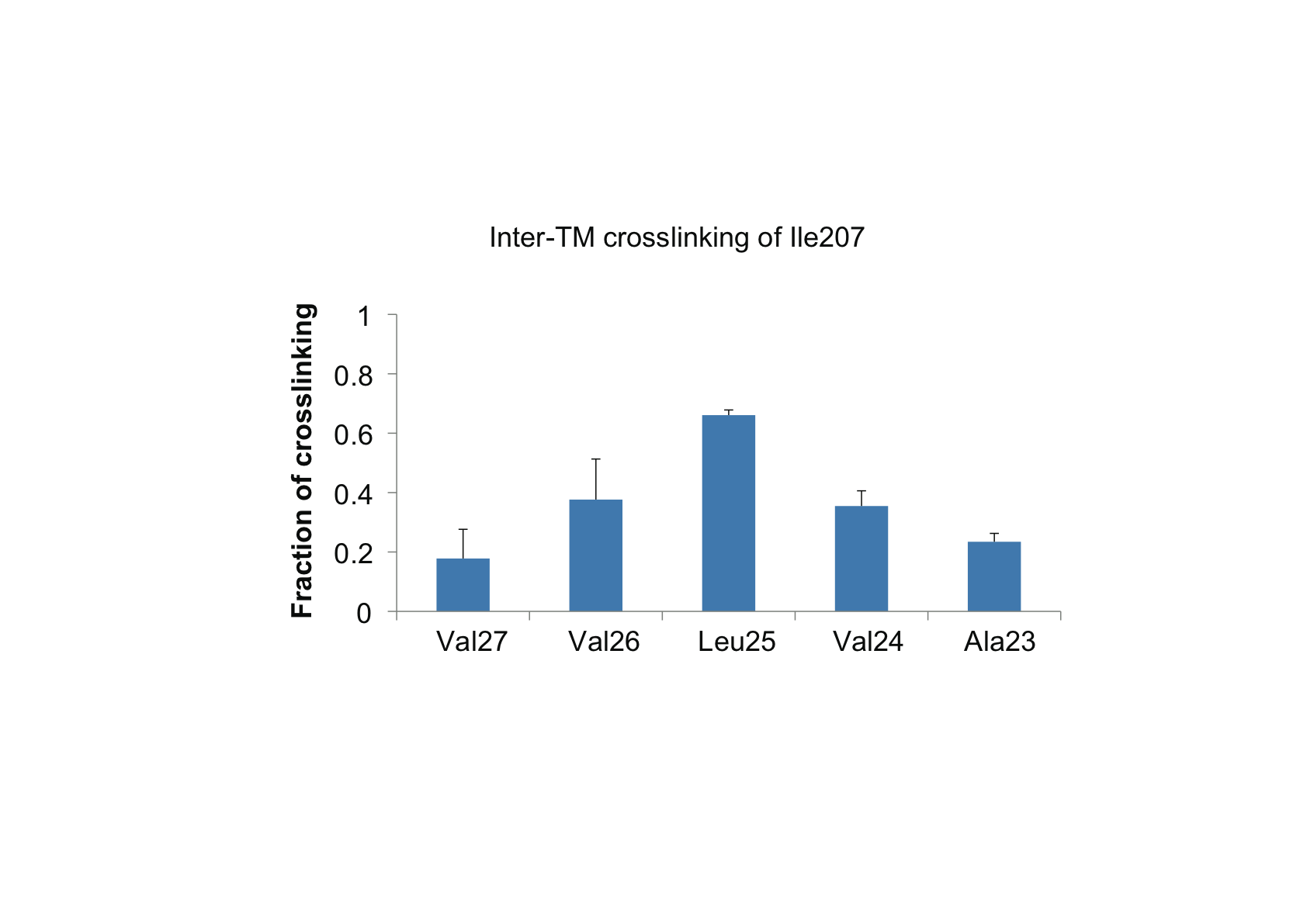

Supplement: Figure S3 — Inter-TM crosslinking for Ile207. Results of inter-TM crosslinking experiment between residue 207 on TM2 and a window of TM1 residues between 23 and 27. Error bars represent two independent experiments. (TIFF) [file pcbi.1002878.s003.tiff]

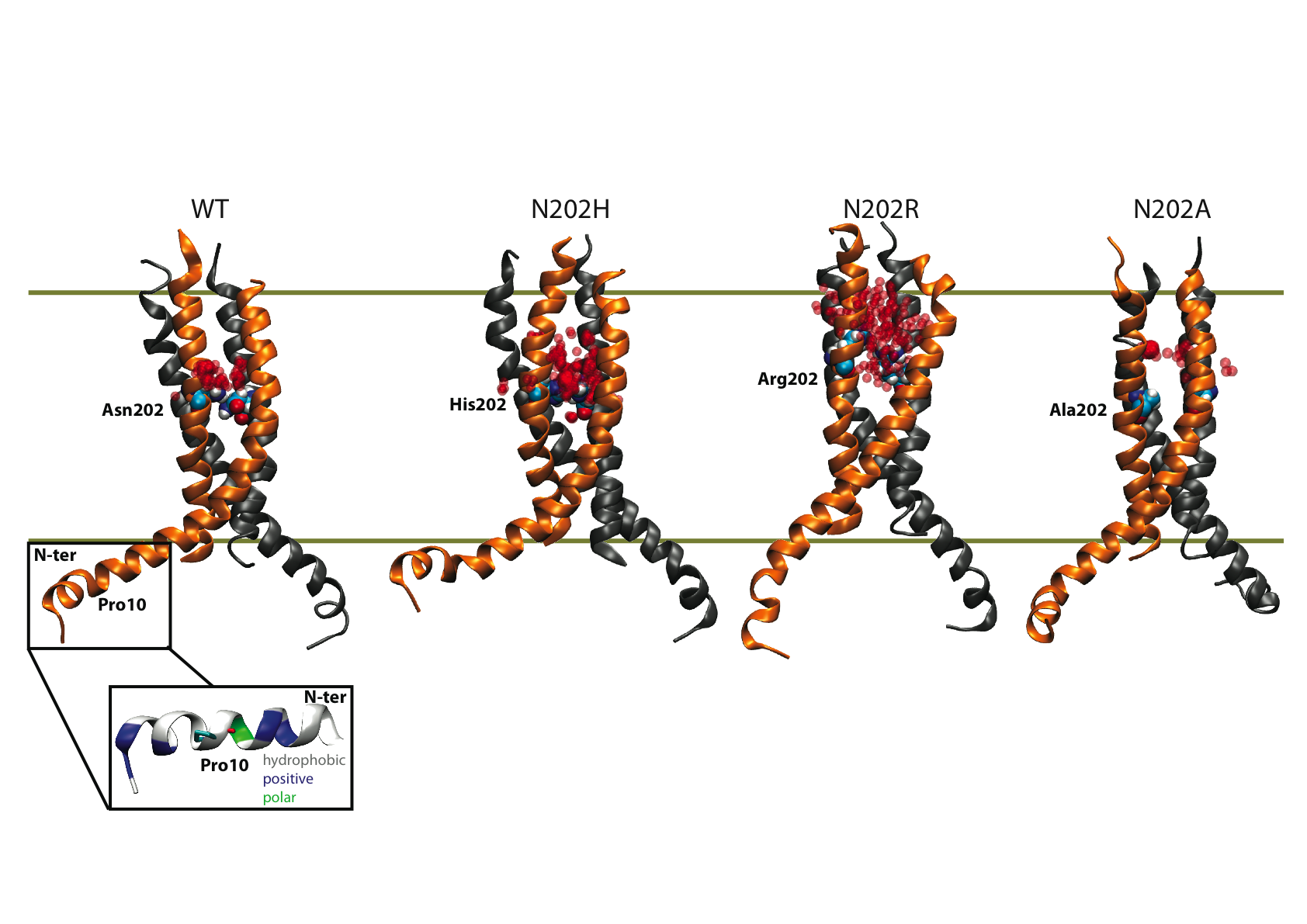

Supplement: Figure S4 — Water distribution around the TM bundle. (Top) The water molecules in contact with the TM bundle for wild type (WT), and three mutants: N202H, N202R and N202A are displayed with red spheres (see also Figure 4 in the main text). (Bottom) The focus on the N-terminal helix extended from TM1 illustrates its amphiphilic nature in our model. The hydrophobic residues are displayed in white, the polar and positively charged residue in green and blue, respectively. (TIFF) [file pcbi.1002878.s004.tiff]
