# Supplementary material for: Assembly of the Transmembrane Domain of E. coli PhoQ Histidine Kinase: Implications for Signal Transduction from Molecular Simulations
Source: PLoS Comput Biol. 2013 Jan 24;9(1):e1002878. doi: 10.1371/journal.pcbi.1002878 (PMC3554529; doi:10.1371/journal.pcbi.1002878)
Supplement: Table S1 — Transmembrane domain predictions. The transmembrane domain considered for the modeling is highlighted in grey as a consensus obtained from the following programs: SPOCTOPUS [59], OCTOPUS [60], PSIpred [49], TMHMM [61], HMMTOP [62], TMpred [63], topcons single [64], and DAS [65]. (PDF) [file pcbi.1002878.s005.pdf]

TM1 (21–42)

Sequence	Method
FLLATAAVVLVLSLAYGMVAL	uniprot
FLLATAAVVLVLSLAYGMVALIG	HMMTOP
ATAAVVLVLSLAYGMVALIGYSV	TMHMM
FLLATAAVVLVLSLAYGMVALIGY	TMpred
FLLATAAVVLVLSLAYGMVALIG	Octopus
FLLATAAVVLVLSLAYGMV	Soctopus
FLLATAAVVLVLSLAYGMVALIGY	psired
FLLATAAVVLVLSLAYGMVAL	topcons single
FLLATAAVVLVLSLAYGMVAL	DAS

TM2 (195–215)

Sequence	Method
FIYVLSANLLLVIPLLWVAAW	uniprot
VWSWFIYVLSANLLLVIPLLWVAA	HMMTOP
WFIYVLSANLLLVIPLLWVAAWW	TMHMM
FIYVLSANLLLVIPLLWVAAW	TMpred
SWFIYVLSANLLLVIPLLWVA	Octopus
SWFIYVLSANLLLVIPLLWVA	Soctopus
FIYVLSANLLLVIPLLWVAAW	psipred
FIYVLSANLLLVIPLLWVAAWW	topcons single
FIYVLSANLLLVIPLLWVA	DAS
