# Supplementary material for: Assembly of the Transmembrane Domain of E. coli PhoQ Histidine Kinase: Implications for Signal Transduction from Molecular Simulations
Source: PLoS Comput Biol. 2013 Jan 24;9(1):e1002878. doi: 10.1371/journal.pcbi.1002878 (PMC3554529; doi:10.1371/journal.pcbi.1002878)
Supplement: Table S2 — List of performed simulations. (PDF) [file pcbi.1002878.s006.pdf]

System	Membrane	Number of atoms	Simulation time
TM1 monomer	POPC	27,333	50 ns
TM2 monomer	POPC	27,956	50 ns
Dimerization TM1	DODE	40,700	100 ns
Dimerization TM2	DODE	40,700	100 ns
Dimerization TM1-TM2	DODE	40,700	100 ns
Initial tetramer	DODE	40,000	60 ns
Initial tetramer	POPE	47,300	80 ns
Extended tetramer	POPE	51,200	150 ns
N202A	POPE	51,200	100 ns
N202H	POPE	51,200	100 ns
N202R	POPE	51,200	100 ns
Metadynamics	POPE	51,200	3x30ns
