# Supplementary material for: Assembly of the Transmembrane Domain of E. coli PhoQ Histidine Kinase: Implications for Signal Transduction from Molecular Simulations
Source: PLoS Comput Biol. 2013 Jan 24;9(1):e1002878. doi: 10.1371/journal.pcbi.1002878 (PMC3554529; doi:10.1371/journal.pcbi.1002878)
Supplement: Text S1 — Supporting methods and materials. Complementary details on the molecular dynamics simulation and cross-linking experiments. (PDF) [file pcbi.1002878.s007.pdf]

Supplementary Methods

Molecular dynamics setup. All simulations were performed using NAMD [1] engine, with the CHARMM27 force field [2], including CMAP corrections. TIP3P water [3] parameterization was used to describe the water molecules. The spatial overlapping of lipid molecules and protein were removed and the resulting protein-membrane system was solvated in a variable-sized water box, neutralized through the addition of NaCl at a concentration of 150 mM. The periodic electrostatic interactions were computed using particle-mesh Ewald (PME) summation with a grid spacing smaller then 1 Å. All systems were first minimized by 2000 conjugate gradient steps. POPE membranes were equilibrated for 500 ps at 300 K with a restraint on the proteins, water and ions. Since the DODE membrane was already equilibrated, this step was not required. All systems were subsequently gradually headed from 0 to 300 K in 800 ps with a constraint on the protein backbone scaffold. Finally, the systems were equilibrated for 3 ns at 300 K. Free molecular dynamics of all equilibrated systems were performed with a 2 fs integration time step using the RATTLE algorithm applied to all bonds. Constant temperature (300 K) was imposed by using Langevin dynamics [4], with a damping coefficient of 1.0 ps. A constant pressure of 1 atm was maintained with a Langevin piston dynamics [5], 200 fs decay period and 50 fs time constant.

Crosslinking reactions, Western blotting and analysis. Oxidized membranes were reconstituted in 20uL of loading buffer (Invitorgen LDS buffer plus 8M urea and 0.5M NEM) and heated for 10 minutes at 70 degrees C. 5uL of sample were loaded onto either a 7% or 3-8% gradient Tris Actetate gel (NuPage, Invitrogen). Proteins were separated by molecular weight with application of 150V for 60 minutes. Proteins were transferred to a nitrocellulose membrane by electrophoresis (iBlot, Invitrogen). Membranes were washed with Tbst (10mM tris pH 7.5, 2.5 mM EDTA, 50 mM NaCl, 0.1% Tween 20) and blocked with 3% BSA in Tbst. PhoQ* was probed using Qiagen penta-his antibody. His antibody was probed with HRP conjugated sheep anti mouse IgG. Proteins were visualized by exposure to ECL reagent (Amersham, GE health sciences) for 1 minute and exposure to film for 30 – 60 seconds. Pixel density histograms were generated using the ImageJ software, freely available from NIH (http://rsbweb.nih.gov/ij/), and crosslinking efficiency was determined using the fraction of cross-linked dimer to total visible protein (dimer/dimer+monomer).

1. Phillips JC, Braun R, Wang W, Gumbart J, Tajkhorshid E, et al. (2005) Scalable molecular dynamics with NAMD. Journal of Computational Chemistry 26: 1781-1802.
2. Brooks BR, Brooks CL, 3rd, Mackerell AD, Jr., Nilsson L, Petrella RJ, et al. (2009) CHARMM: the biomolecular simulation program. J Comput Chem 30: 1545-1614.
3. Jorgensen WL, Chandrasekhar J, Madura JD, Impey RW, Klein ML (1983) Comparison of simple potential functions for simulating liquid water. The Journal of Chemical Physics 79: 926.
4. Brunger A, Brooks CL (1984) Stochastic boundary conditions for molecular dynamics simulations of ST2 water. Chemical Physics Letters 105: 495-500.
5. Feller SE, Zhang Y, Pastor RW, Brooks BR (1995) Constant pressure molecular dynamics simulation: the Langevin piston method. The Journal of Chemical Physics 103: 4613.
